# RNAi Screen Identifies Novel Regulators of RNP Granules in the *Caenorhabditis elegans* Germ Line

**DOI:** 10.1534/g3.116.031559

**Published:** 2016-06-09

**Authors:** Megan P. Wood, Angela Hollis, Ashley L. Severance, Megan L. Karrick, Jennifer A. Schisa

**Affiliations:** Department of Biology, Central Michigan University, Mount Pleasant, Michigan 48859

**Keywords:** RNP granule, oocyte quality, *C. elegans*, aging, germ line

## Abstract

Complexes of RNA and RNA binding proteins form large-scale supramolecular structures under many cellular contexts. In *Caenorhabditis elegans*, small germ granules are present in the germ line that share characteristics with liquid droplets that undergo phase transitions. In meiotically-arrested oocytes of middle-aged hermaphrodites, the germ granules appear to aggregate or condense into large assemblies of RNA-binding proteins and maternal mRNAs. Prior characterization of the assembly of large-scale RNP structures via candidate approaches has identified a small number of regulators of phase transitions in the *C. elegans* germ line; however, the assembly, function, and regulation of these large RNP assemblies remain incompletely understood. To identify genes that promote remodeling and assembly of large RNP granules in meiotically-arrested oocytes, we performed a targeted, functional RNAi screen and identified over 300 genes that regulate the assembly of the RNA-binding protein MEX-3 into large granules. Among the most common GO classes are several categories related to RNA biology, as well as novel categories such as cell cortex, ER, and chromosome segregation. We found that arrested oocytes that fail to localize MEX-3 into cortical granules display reduced oocyte quality, consistent with the idea that the larger RNP assemblies promote oocyte quality when fertilization is delayed. Interestingly, a relatively small number of genes overlap with the regulators of germ granule assembly during normal development, or with the regulators of solid RNP granules in *cgh-1* oocytes, suggesting fundamental differences in the regulation of RNP granule phase transitions during meiotic arrest.

RNP granules are dynamic, membrane-free organelles in the cytoplasm of diverse cell types of plants, animals, and fungi. These ribonucleoprotein complexes are composed of RNA and RNA-binding proteins, and have roles in the regulation of mRNA transport, decay, translation, and storage. Examples of RNP granules include processing bodies, stress granules, neuronal granules, and germ granules that are unique to the germ line. Germ granules are found in a range of invertebrates and vertebrates, and are proposed to have roles in promoting germ cell fate and function since mutations in the components’ genes often result in sterility (Gruidl *et al.* 1996; Kawasaki *et al.* 1998). The composition, size, and subcellular localization of germ granules are dynamic, and remodeling of the RNPs occurs throughout development (Schisa 2014; Sheth *et al.* 2010; Voronina 2013).

Evidence from several developmental systems supports multiple RNP granule assembly pathways. A major role for RNA biogenesis in regulating the structure, size, and organization of germ granules comes from studies in flies, mice, frogs, and worms (Soderstrom and Parvinen 1976; Delanoue *et al.* 2007; Updike and Strome 2009; Kloc and Etkin 1994). Nucleation of RNP granules by key “seed” proteins is suggested by studies in zebrafish and flies, where Buckyball and *oskar* are necessary and sufficient to induce the assembly of germ granules (Bontems *et al.* 2009; Marlow and Mullins 2008; Mahowald 2001). Similarly, in worms, PGL-1 is sufficient to promote formation of ectopic cytoplasmic germ granules (Hanazawa *et al.* 2011; Updike *et al.* 2011).

The large-scale organization of RNP complexes has been investigated using a combination of *in vitro* and high-resolution imaging approaches (Weber and Brangwynne 2012; Kato *et al.* 2012; Wang *et al.* 2014; Lin *et al.* 2015). Many of the RNA-binding proteins that are enriched in RNP granules contain intrinsically disordered regions (IDRs), and these sequences appear to contribute to the formation of RNP granules in several systems (Gilks *et al.* 2004; Decker *et al.* 2007; Lee *et al.* 2013; Updike *et al.* 2011; Wang *et al.* 2014; Elbaum-Garfinkle *et al.* 2015). *In vitro* studies suggest that IDRs may promote granule formation via the formation of fiber-containing hydrogels (Kato *et al.* 2012; Han *et al.* 2012), and certain proteins appear to undergo liquid–liquid phase separation (LLPS) in a salt- and temperature-dependent manner (Yeo *et al.* 2011; Nott *et al.* 2015). LLPS describes the nonmembrane compartments in cells that form by phase separation from the cytoplasm (Hyman *et al.* 2014). A unified model suggests that a progression from dynamic liquid to more stable fibers in cells may result in RNP structures with varying physical and chemical properties, depending on the biological state of the cell (Lin *et al.* 2015).

Germ granules in *Caenorhabditis elegans* embryos and oocytes (also referred to as P granules) share characteristics with liquid droplets that can undergo phase transitions (Brangwynne *et al.* 2009, 2011; Hubstenberger *et al.* 2013). In the oocytes of young hermaphrodites, meiotic maturation is active, and RNP components are in mobile, small particles, including germ granules and processing (P) bodies, that are diffuse throughout the cytoplasm (McCarter *et al.* 1999; Hubstenberger *et al.* 2013). In contrast, in meiotically-arrested oocytes, the germ granules appear to aggregate into large assemblies of RNA-binding proteins and maternal mRNAs (Figure 1A; Schisa *et al.* 2001; Jud *et al.* 2008; Noble *et al.* 2008). These condensed RNP assemblies maintain characteristics of liquid droplets, like the smaller particles; however, the largest RNP granules have slower dynamics than the smaller RNP granules (Hubstenberger *et al.* 2013). Initial characterization of the assembly of large-scale, supramolecular structures via candidate approaches has identified a small number of regulators of RNP phase transitions in the *C. elegans* germ line, including CAR-1, PUF-5, PUF-3, GLH-1, DCR-1, and CEH-18 (Noble *et al.* 2008; Jud *et al.* 2008; Beshore *et al.* 2011; Hubstenberger *et al.* 2013). However, the assembly, function, and regulation of these large RNP assemblies remain incompletely understood.

**Figure 1:**
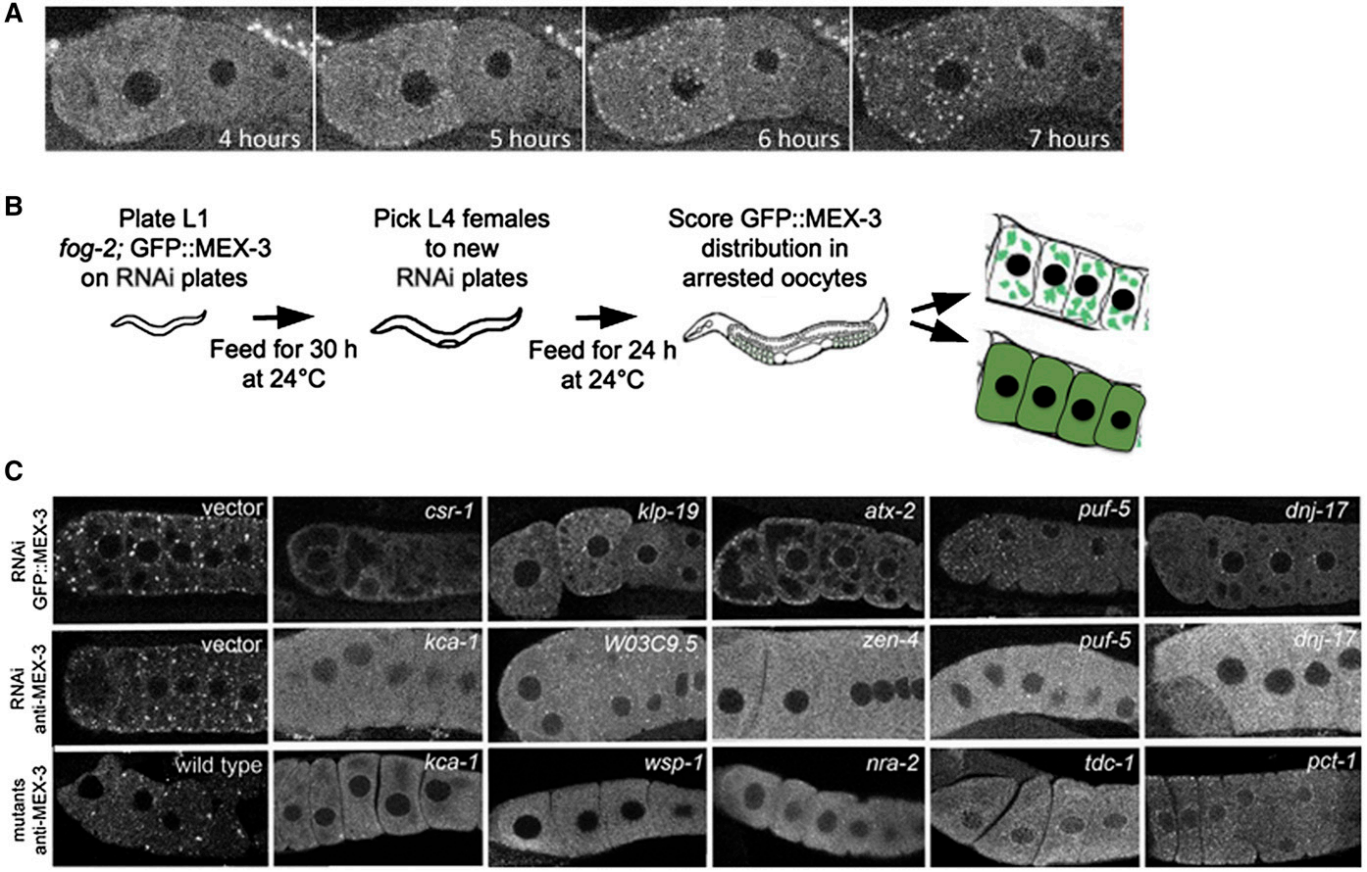
RNAi screen identifies positive regulators of RNP granule assembly in arrested oocytes. (A) Live imaging of *fog-2*;GFP::MEX-3 unmated female shows rapid assembly of GFP::MEX-3 granules begins soon after the L4 stage of development. By 7 hr post-L4, granules are prominent and enriched near the cortex and nuclear membrane, and the level of cytosolic GFP is decreased relative to 4 hr post-L4. (B) Cartoon of feeding RNAi screen. 1536 genes were screened and 319 genes were identified. (C) Representative images showing disruption of RNP granule assembly after knockdown of gene expression. Top row: GFP::MEX-3 distribution after RNAi in *fog-2* background. The negative control was RNAi using an empty vector (left), and GFP::MEX-3 granules are prominent. RNP granules are disrupted, and levels of diffuse GFP::MEX-3 are increased after knockdown of five positive hits. Middle row: Anti-MEX-3 staining after RNAi in *fog-2* worms reveals similar large granules in negative control (left) and similar disruption of endogenous RNP granules. Bottom row: Anti-MEX-3 staining in arrested oocytes of wild-type (left) and in mutants depleted of sperm validates the GFP::MEX-3 reporter results. GFP, Green fluorescent protein; RNAi, RNA interference; RNP, ribonucleoprotein.

To identify genes that promote remodeling and assembly of large RNP granules in meiotically-arrested oocytes, we performed a targeted, functional RNAi screen, and identified over 300 genes that regulate the assembly of the RNA-binding protein MEX-3 into large granules in arrested oocytes (Figure 1A). Among the most common GO classes are several categories related to RNA biology, as well as some unexpected categories such as chromosome segregation. Interestingly, a relatively small number of genes overlap with the regulators of germ granule assembly identified during normal development or with the modifiers of solid RNP granules in oocytes (Updike and Strome 2009; Wang *et al.* 2014; Elbaum-Garfinkle *et al.* 2015; Hubstenberger *et al.* 2015), suggesting fundamental differences in the regulation of RNP granule phase transitions during meiotic arrest.

## Materials and Methods

### Strains and culture

Worm cultures were maintained on NGM plates at 20° or 24° (Brenner 1974). The following transgenic strain, *P_pie-1_*:: GFP::MEX-3::UTR*^pie-1^*, was crossed into *fog-2(q71)* (Jud *et al.* 2008), and L4 females were segregated from males and grown at 24° for 1 d prior to scoring arrested oocytes in females. Strains are available upon request.

### RNAi screen

Day 1: Clones representing the oogenesis-enriched or germ line intrinsic genes (Reinke *et al.* 2004) were streaked onto LB plates with carbenicillin and incubated at 37° overnight. The L4440 vector was the negative control; *inx-14* was a positive control for inhibiting GFP::MEX-3 granule assembly. Twelve-well RNAi plates contained carbenicillin and IPTG (Kamath and Ahringer 2003). Day 3: Bacterial cultures were grown in LB and carbenicillin for 6–12 hr at 37°. Day 4: 12-well RNAi plates were seeded with RNAi bacteria. Day 5: Gravid *fog-2*; GFP::MEX-3 adults were bleached, and embryos were incubated in M9 at 20° overnight. Day 6: ∼20–30 synchronized L1 *fog-2*; GFP::MEX-3 worms were plated into each well. Day 7: L4 males were removed from each well. Day 8: Between 10–15 unmated females were scored 1 d post-L4 using a Leica MZ16F or Olympus BX51 equipped with epifluorescence. Worms were primarily scored for the assembly of large RNP granules in proximal oocytes. In the negative control (vector RNAi), large GFP::MEX-3 granules were observed 100% of the time. Worms in which the GFP::MEX-3 was diffusely cytoplasmic, with no detectable granules, were scored as “Diffuse.” Intermediate phenotypes were also observed in which RNAi depletions induced diffuse GFP::MEX-3 combined with small GFP::MEX-3 granules; this phenotype is indicated as “Sm” for small granules in Supplemental Material, Table S1. Representative images were collected using the Nikon A1R confocal microscope and formatted using Adobe Photoshop CS5. A minimum of three replicates were performed for positive hits. A subset of RNAi phenotypes was validated by assaying endogenous MEX-3 (*n* > 20 worms per gene; see fluorescence microscopy), and/or by scoring RNP granule phenotypes in arrested oocytes of corresponding deletion alleles: *wsp-1(gm324)*, *kca-1(ok2777)*, *nra-2(ok1731)*, *tdc-1(ok914)*, and *pct-1(ok1348*). Gene ontology classes were determined using PANTHER (release 20150430); the annotation data sets were experimental only, and the version was the GO Ontology database released 2015-08-06 (Mi *et al.* 2016).

### Fluorescence microscopy

Antibodies, antisera, and staining protocols were as described: anti-MEX-3 (Draper *et al.* 1996) (antibody from Dr. James Priess); anti-CGH-1 C [fixation as in Jud *et al.* (2008), antibody from Dr. David Greenstein]; anti-GLH-1 [fixation as in Beshore *et al.* (2011)]. Secondary antibodies were from Molecular Probes. Images were collected using a Nikon A1R laser scanning confocal microscope and analyzed using Adobe Photoshop or Image J.

### Oocyte viability assay

To determine the viability of arrested oocytes, the GFP::MEX-3; *fog-2* strain was used (Jud *et al.* 2008). After RNAi, the number of oocytes accumulated in each gonad arm was counted using DIC optics, and GFP distribution was examined (either granules failed to assemble or they had assembled). Matings were set with individual females and *fog-2(q71)* males, which are fertile (Schedl and Kimble 1988). When the total number of unfertilized oocytes, embryos, and larvae laid on the plate was equal to the number of accumulated oocytes prior to mating, adults were removed from the plate. The viability of the embryos was determined 24 hr later by counting the number of hatched larvae *vs.* the number of unhatched embryos.

### Data availability

The authors state that all data necessary for confirming the conclusions presented in the article are represented fully within the article.

## Results and Discussion

### Diverse regulators of RNP granule assembly

To systematically identify proteins involved in oocyte RNP assembly, we carried out a functional RNA-mediated interference (RNAi) screen using the Ahringer and Vidal RNAi feeding libraries (Kamath and Ahringer 2003; Rual *et al.* 2004). We screened 1536 genes, a subset of the genes classified as “oogenesis enriched” or “germ line intrinsic” by microarray analyses (Reinke *et al.* 2004), and looked for a failure of MEX-3 granule assembly in arrested oocytes, using the *fog-2*;GFP::MEX-3 strain (Figure 1B). In *fog-2* hermaphrodites, no sperm are made; therefore, the worms are functionally females with meiotically-arrested oocytes (Schedl and Kimble 1988). MEX-3 was an effective marker since it is diffusely distributed throughout the cytoplasm of active oocytes, but is highly enriched in granules in arrested oocytes, with much lower levels diffuse throughout the cytoplasm. In 100% of control RNAi worms, large GFP::MEX-3 granules were detected in all arrested oocytes (*n* > 100, Figure 1C). We identified 319 genes that we first subdivided into strong, intermediate, and weak positives based on the penetrance of the phenotype (Table S1). The identity of all *E. coli* RNAi clones that produced a phenotype was verified by DNA sequencing. Among the positives, 112 were classified as strong regulators, with > 75% penetrance of failure to assemble MEX-3 into large granules in arrested oocytes (Table S1, green genes). 106 genes were classified as intermediate regulators, with 50–74% penetrance (blue genes); and 101 genes were classified as weak regulators, with 10–49% penetrance (Table S1, red genes). Among all classes we saw a range of phenotypes, from oocytes with GFP::MEX-3 distributed at high levels diffusely throughout the cytoplasm, and sometimes in small granules near the nuclear envelope, but not enriched in any large cortical granules (*e.g.*, *dnj-17*, Figure 1C, indicated as D in Table S1), to oocytes with diffuse MEX-3 as well as small, but not large, granules (*e.g.*, *csr-1* and *atx-2*, Figure 1C, indicated as Sm in Table S1). We also observed some phenotypes with small GFP granules in the oldest, most proximal oocytes but diffuse GFP in the more distal oocytes (*e.g.*, *puf-5*). We validated the GFP-based screen by scoring endogenous MEX-3 after RNAi for a subset of the positives and found no false positives among the 43 genes scored (Figure 1C middle row; Table S1). For each gene, the phenotype penetrance was at least as high as that scored using GFP::MEX-3. The identification of expected positives including *puf-5*, *dcr-1*, *car-1*, and *ceh-18* also validated our screen (Jud *et al.* 2008; Noble *et al.* 2008; Beshore *et al.* 2011). In addition, we scored the distribution of endogenous MEX-3 in the arrested oocytes of several deletion alleles: *wsp-1(gm324)*, *kca-1(ok2777)*, *nra-2(ok1731)*, *tdc-1(ok914)*, and *pct-1(ok1348*). In the control, wild-type hermaphrodites depleted of sperm with meiotically-arrested oocytes, large MEX-3 granules were observed in 100% of arrested oocytes (Figure 1C, bottom row). In all five deletion alleles, we observed high levels of diffuse MEX-3 staining throughout the cytoplasm (> 50% germ lines; *n* > 25). In most cases, MEX-3 granules were not detected, although for *pct-1*, small granules were sometimes detected (Figure 1C, bottom row). This screen, like most genetic screens, also had false negatives and did not uncover all previously identified regulators of RNP granule assembly, *e.g.*, *puf-3* (Hubstenberger *et al.* 2013). One limitation of our screen design was the exposure of worms to RNAi bacteria starting at the L1 stage; therefore, genes required for early germ line development could have been missed. Since we did not quantitate meiotic arrest directly, one caveat to our results is that the disruption of RNP granule assembly in some cases may be due to indirect effects. However, for the majority of gene knockdowns, oocyte morphology and maturation lacked obvious defects. Another consideration is that altered levels of RNP granule components or other changes in mRNA metabolism may contribute to the observed phenotypes. Nevertheless, our results should provide a foundation for future analyses to resolve these questions and clarify the roles of these protein classes in the regulation and function of RNP complexes in the germ line.

The genes we identified span a large number of molecular functions, cellular compartments, and biological processes, and the majority of the genes have human orthologs (Table S1) (Shaye and Greenwald 2011). Some of the GO cell component classes with large numbers of genes were related to RNA biology, for example “RNP granule;” while other GO classes such as “nuclear chromosome” were novel (Table 1). Some of the GO biological process classes were expected, including oogenesis and germ line sex determination. In contrast, the categories of meiotic chromosome segregation, RNA interference, and cell cycle were not anticipated. The majority of the GO classes of the positive hits are also generally enriched among the oogenesis-enriched and germ line-enriched genes that were screened. However, the converse is not true, as only a subset of GO classes enriched among germ line genes were identified among the positives. Below, we discuss several major categories of genes associated with the regulation of RNP remodeling.

**Table 1:** GO annotation

GO Class Description	# Genes in Genome	# Hits	Genes*^a^*
Germ plasm	58	7	*mex-3*, *car-1*, *puf-5*, *pgl-2*, *pie-1*, *csr-1*, *ife-1*, *cacn-1*
Pole plasm	58	7	
P granule	58	7	
Cytoplasmic ribonucleoprotein granule	64	7	
Ribonucleoprotein granule	65	7	
Ribonucleoprotein complex	78	8	
Cell cortex	75	6	*rga-3*, *srgp-1*, *kin-18*, *wsp-1*, *cav-1*, *sao-1*
Nuclear chromosome	62	6	*ekl-1*, *csr-1*, *set-25*, *zhp-3*, *ceh-39*, *klp-19*
Condensed nuclear chromosome	40	5	
Meiotic chromosome segregation	137	14	*tbg-1*, *zen-4*, *cyp-31A3*, *cav-1*, *srgp-1*, *kca-1*, *klp-19*, *ooc-3*, *car-1*, *zhp-3*, *arp-6*, *fbxa-10*, *hpo-9*, *rad-50*, *him-10*, *set-2*, *coh-3*, *eri-1*, *dcr-1*, *atl-1*, *scc-1*, *spo-11*, *atx-2*, *cpg-2*, *mpk-1*, *cki-2*, C18H2.2
Nuclear chromosome segregation	156	15	
Chromosome segregation	169	15	
Meiotic nuclear division	212	20	
Meiotic cell cycle process	218	20	
Meiotic cell cycle	219	20	
Regulation of cell cycle	190	12	
Cell cycle	425	28	
Cell cycle process	420	28	
Nuclear division	269	24	
Organelle fission	270	24	
RNA interference	136	12	*ekl-1*, *ceh-39*, *eri-1*, *dcr-1*, *sams-3*, *rpn-10*, *cpsf-2*, *cin-4*, *pro-2*, *tftc-5*, *mex-3*, *pie-1*, *phf-30*, *sti-1*, CO4F12.1, W06E11.1
Gene silencing by RNA	141	12	
Post-transcriptional gene silencing by RNA	141	12	
Gene silencing	141	12	
Post-transcriptional gene silencing	141	12	
Post-transcriptional regulation of gene expression	161	13	
Regulation of gene expression, epigenetic	160	12	
Negative regulation of gene expression	209	15	
Negative regulation of macromolecule Metabolic process	235	15	
Negative regulation of metabolic process	239	16	
Hermaphrodite sex determination	48	7	*rpn-10*, *ceh-39*, *atx-2*, *cacn-1*, *fem-3*, *ddx-23*, *rnp-4*, C07A9.2
Sex determination	60	8	

GO, Gene Ontology; #, number.

a Genes listed for each set of related GO classes.

#### Genes associated with the germ (or pole) plasm, P granules, and RNP granules:

This category of eight genes includes: *mex-3*, *car-1*, *puf-5*, *pgl-2*, *pie-1*, *csr-1*, *ife-1*, and *cacn-1* (Figure 1C and Table 1). Three genes encode characterized components of the RNP granules, MEX-3, CAR-1, and PUF-5 (Jud *et al.* 2008; Noble *et al.* 2008). While *car-1* and *puf-5* were previously identified as regulators of RNP granules (Noble *et al.* 2008), *mex-3* is a novel regulator. PGL-2 and PIE-1 localize to germ granules during postembryonic development but not in the adult germ line; PGL-2 is a novel protein, and PIE-1 is a transcriptional and translational regulator (Kawasaki *et al.* 2004; Mello *et al.* 1996; Tenenhaus *et al.* 1998). While it is not known if PGL-2 or PIE-1 localize to large RNP granules, a subset of germ granule proteins do, including PGL-1 and GLH-1 (Schisa *et al.* 2001). Two proteins in this category, CSR-1 and IFE-1, localize to germ granules in the adult germ line (Claycomb *et al.* 2009; Amiri *et al.* 2001), and *csr-1* is required for their proper assembly (Updike and Strome 2009). These results support the idea that a subset of germ granule proteins may function to “seed” the assembly of large RNP granules in arrested oocytes. The last protein, CACN-1, appears to be a component of the spliceosome; it has functions in regulating the sperm/oocyte switch in hermaphrodites and is speculated to regulate targets post-transcriptionally (Doherty *et al.* 2014). Although a link between sex determination genes and RNP remodeling is not obvious, a recent study of the sex determination gene *laf-1* uncovered a role for LAF-1 in promoting the assembly of germ granules in the embryo (Elbaum-Garfinkle *et al.* 2015; Hubert and Anderson 2009).

#### Genes associated with the cell cortex:

Six genes were identified in the cell cortex GO class: *rga-3*, *sao-1*, *cav-1*, *srgp-1*, *kin-18*, and *wsp-1* (Figure 1C and Table 1). Although the large RNP granules are cortically enriched within oocytes, none of these genes have been implicated as regulators of RNP assembly or condensation. Interestingly, three genes function in regulating the Rho family of small GTPases. *rga-3*, along with *rga-4*, controls the activity of RHO-1 and contractility of the acto-myosin network (Schmutz *et al.* 2007; Schonegg *et al.* 2007). *kin-18* is a Tao/Ste20-like kinase that also regulates cortical contractility in a RHO-dependent manner, and has provided a link between cytoskeleton remodeling and cell polarity establishment (Spiga *et al.* 2013). Lastly, *srgp-1* is a RhoGAP with roles in cell clearance during programmed cell death and in modifying chromosome morphogenesis (Neukomm *et al.* 2011; Colaiacovo *et al.* 2002). Since Rho GTPases can interact with a large and diverse number of downstream targets (Ridley 2001), the precise way they influence RNP remodeling is not clear; however, their roles in regulating the actin cytoskeleton are intriguing given the number of cytoskeleton genes identified in the screen. The cell cortex gene *wsp-1*, for example, colocalizes with actin at cell boundaries and activates the Arp2/3 complex that affects actin nucleation and branching (Sawa *et al.* 2003; Lundquist 2006). Additional genes in the cell cortex category include *cav-1*, which regulates progression through the meiotic cell cycle, suggesting it likely has an indirect role in regulating RNP remodeling (Govindan *et al.* 2009), and *sao-1* which encodes a GYF domain-containing protein that functions with *sel-10* to negatively regulate the Notch receptor signaling pathway in the embryo (Hale *et al.* 2012).

#### Genes associated with the cytoskeleton and ER:

When we examined the complete list of cell component GO classes ordered by number of genes, we noticed that eight genes were associated with the cytoskeleton class, including two that are in the cell cortex group, *rga-3* and *wsp-1*. Three of the other six genes are kinesins, possibly implicating plus-end directed, microtubule motor proteins as important regulators promoting the condensation of RNP granule components into granules (*kca-1*, *zen-4*, and *klp-19*, Figure 1C). We had speculated that microtubules might mediate the dynamics of RNP granules based on published studies. First, α- and β-tubulin become cortically enriched in arrested oocytes (Harris *et al.* 2006). The redistribution of the microtubules mirrors the redistribution of MEX-3 protein, from being diffusely cytoplasmic to being enriched at the cortex and near the nuclear membrane (Figure 1A). Second, the disruption of microtubules affects the assembly of stress granules and processing (P) bodies (Ivanov *et al.* 2003; Sweet *et al.* 2007; Aizer *et al.* 2008). The first kinesin, *kca-1*, encodes a kinesin cargo adaptor that localizes to the Kinesin-1 complex (Yang *et al.* 2005). Loss of function of the Kinesin-1 complex interferes with the translocation of meiosis metaphase I spindles to the *C. elegans* oocyte cortex. Additional roles for Kinesin-1 are seen in the transport of RNP granules in *Drosophila* neurons (Ling *et al.* 2004). For *zen-4* and *klp-19*, no functions in transporting cargo in the oocyte cytoplasm have been demonstrated. *zen-4* encodes a kinesin-like protein in the kinesin-6 subfamily of plus-end-directed microtubule motors (Raich *et al.* 1998) and, with the RhoGAP CYK-4, ZEN-4 forms the centralspindlin complex that functions late in anaphase and during cytokinesis in early embryos (Hizlan *et al.* 2006). *klp-19* encodes a motor related to members of the kinesin-4 family, and it localizes to the nucleoplasm of nuclei in the distal germ line and to late prophase chromosomes (Powers *et al.* 2004). *tbg-1* encodes γ-tubulin, which localizes to centrosomes of mitotic germ nuclei, and to the nuclear envelope of maturing oocytes (Bobinnec *et al.* 2000; McNally *et al.* 2006). γ-tubulin plays critical roles in nucleating microtubules and in the MTOC. In *Drosophila*, components of the γ-tubulin ring complex are required for localization of *bicoid* RNA to the anterior of the oocyte (Schnorrer *et al.* 2002). The final two genes, *car-1* and *pie-1*, encode RNA-binding proteins that have less well-characterized roles in regulating the cytoskeleton.

The four genes associated with the ER include *car-1*, *ooc-3*, *ufd-1*, and *nra-2* (Figure 1C and Table 1). Genes associated with the ER were intriguing because RNP granules associate with the ER in oocytes of many species (Schisa 2012), and reorganization of the ER is observed in meiotically-arrested *C. elegans* oocytes (Patterson *et al.* 2011). CAR-1 encodes an RNA-binding protein that localizes to the ER in early embryos (Boag *et al.* 2005; Squirrell *et al.* 2006), and its *Drosophila* homolog *Trailerhitch* similarly localizes to the ER in flies (Wilhelm *et al.* 2005). The phenotypes of *car-1* and *Trl* mutants have led to speculation that this conserved RNP complex plays a role in ER organization by regulating protein trafficking through the secretory pathway (Decker and Parker 2006). OOC-3 is a nematode-specific protein that localizes to the ER in the germ line and early embryo, and is required to localize PAR proteins in the two-cell embryo (Basham and Rose 2001; Pichler *et al.* 2000). UFD-1 is part of the Cdc48/Ufd1/Npl4 complex that functions in ER-associated protein degradation (Mouysset *et al.* 2006). The ability of misfolded proteins to be eliminated from the ER is an essential function in adaptation to ER stress. NRA-2 has been characterized as an ER protein that regulates neuronal death (Kamat *et al.* 2014); to date, no function in the germ line has been characterized. It will be interesting to determine if and how the ER is affected in arrested oocytes after knockdown of each of these four genes.

#### Genes associated with condensed nuclear chromosomes:

This set of six genes includes: *ekl-1*, *csr-1*, *set-25*, *zhp-3*, *ceh-39*, and *klp-19* (Figure 1C and Table 1). Three of the six genes, *ekl-1*, *csr-1*, and *set-25*, are regulators of small RNAs, with roles in the 22G RNA pathway. *ekl-1* encodes a Tudor-domain protein that has diverse functions in RNAi, transgene silencing, transgene-mediated cosuppression in the germ line, and chromosome segregation (Kim *et al.* 2005; Robert *et al.* 2005). *csr-1* encodes an Argonaute protein with similarly diverse functions as *ekl*-1 (Grishok *et al.* 2001; Robert *et al.* 2005; Yigit *et al.* 2006). *set-25* encodes a putative histone methyltransferase that may promote a chromatin state favorable for maintenance of nuclear RNAi; it is required for transgenerational RNAi (Ashe *et al.* 2012). Functional links between these small RNA regulators and germ granules have been described. CSR-1 localizes to germ granules as described above, and mutation of either *csr-1* or *ekl-1* disrupts the association of germ granules at the nuclear membrane of germ line nuclei (Vought *et al.* 2005; Claycomb *et al.* 2009). Interestingly, in a screen to identify regulators of germ granules, depletion of *csr-1*, *ego-1*, or *drh-3* resulted in PGL-1 granules of increased size in the germ line and led to a hypothesis that germ granules function as a regulator center for endogenous siRNA silencing in the germ line (Updike and Strome 2009). We did not identify *ego-1* or *drh-3* in our screen, and the *csr-1* phenotype we observed is opposite to the published germ granule phenotype; thus, *csr-1* may have a different function in the germ line when meiotic maturation is arrested. The other three genes in this group, *zhp-3*, *ceh-39*, and *klp-19*, have functions associated with chromosomes in the nucleus, and have not previously been associated with regulating RNP condensation in the cytoplasm; they may act indirectly to regulate the assembly of RNPs.

#### Genes that regulate meiotic chromosome segregation, the cell cycle, or nuclear division:

This large group includes 10 genes in classes described above: *tbg-1* and *zen-4* (cytoskeleton and RNP granules); *cyp-31A3* (RNP granules); *cav-1* and *srgp-1* (cell cortex); *kca-1* and *klp-19* (cytoskeleton); *ooc-3* (ER); *car-1* (RNP granules); *zhp*-3 (nuclear chromosomes); and 17 additional genes: *arp-6*, *fbxa-10*, *hpo-9*, *rad-50*, *him-10*, *set-2*, *coh-3*, *eri-1*, *dcr-1*, *atl-1*, *scc-1*, C18H2.2, *spo-11*, *atx-2*, *cpg-2*, *mpk-1*, and *cki-2* (Figure 1C and Table 1). Of the 17 genes, *dcr-1* and *atx-2* are known regulators of germ granules and/or RNP granules in arrested oocytes (Beshore *et al.* 2011; Updike and Strome 2009). Many of these proteins, similar to the GO class above, seem likely to act indirectly based on the expression and function studies to date. Nonetheless, the large number of genes in this class may suggest novel links between oocyte chromosomes and RNP granule remodeling.

#### Genes that function in post-transcriptional gene silencing by RNA interference or negative regulation of metabolic processes:

This large group includes six genes in classes described above: *ekl-1* and *ceh-39* (nuclear chromosomes), *eri-1* and *dcr-1* (regulate meiotic chromosome segregation); *mex-3* and *pie-1* (germ plasm), and 10 additional genes: *sams-3*, *rpn-10*, *cpsf-2*, *cin-4*, *pro-2*, *tftc-5*, *phf-30*, COFF12.1, W06E11.1, and *sti-1* (Table 1). This category was not a surprise, as at least some untranslated mRNAs are concentrated into RNP granules in a variety of eukaryotes, and functions of RNP granules include the translational control of maternal mRNAs (Decker and Parker 2006).

#### Genes that regulate sex determination:

This group includes four genes in classes described above: *rpn-10*, *ceh-39*, *atx-2*, and *cacn-1* (Figure 1C and Table 1), and four additional genes: *rnp-4*, *ddx-23*, C07A9.2, and *fem-3*. Interestingly, *atx-2* and *rnp-4* are two of the six genes that overlap with the regulators of germ granules, as discussed further below. RNP-4 contains an RRM RNA binding domain, localizes predominantly to nuclei, is required for fertility, and is reported to regulate the formation of P bodies (Sun *et al.* 2011). DDX-23 is required for the sperm/oocyte switch in hermaphrodites, localizes mainly to nuclei, and is speculated to function by modulating RNP complexes (Konishi *et al.* 2008). C07A9.2 functions in germ line development and is an ortholog of the yeast and human splicing factor BUD31. These three genes may indirectly regulate the cytoplasmic RNP complexes, perhaps via the regulation of mRNAs in the nucleus, which is known to affect the translational efficiency of mRNA in the cytoplasm (Matsumoto *et al.* 1998). FEM-3 promotes the male fate similar to the LAF-1 protein, which was recently shown to also function in promoting germ granule formation in the early embryo (Elbaum-Garfinkle *et al.* 2015).

### Decreased oocyte quality correlates with a failure to assemble MEX-3 granules

**Figure 2:**
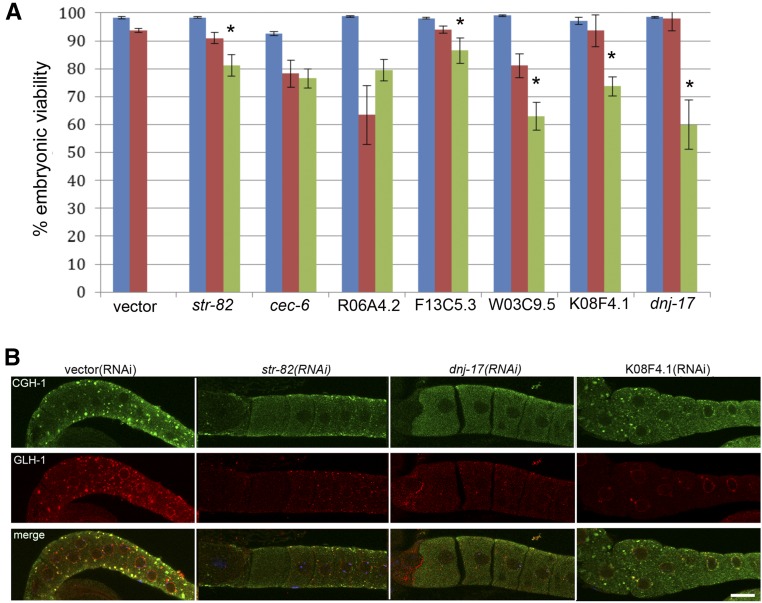
Disruption of RNP granule assembly correlates with decreased oocyte quality. (A) Blue bars show > 90% embryos are viable after RNAi of seven positive hits in wild-type worms, suggesting these genes are reasonable targets for analysis in arrested oocytes. For five of the seven genes, the percent of viable embryos significantly decreased when arrested oocytes did not assemble GFP::MEX-3 granules (green bars), as compared to worms where granules were detected (red bars). The percent embryonic viability is plotted as mean ± SEM; * *P* < 0.05 by Fisher’s exact test or Chi-square test (for W03C9.5, K08F4.1, and *dnj-17*). A minimum of 254 oocytes from a minimum of 11 females were analyzed for each RNAi target. (B) CGH-1 and GLH-1 fail to assemble into cortical granules after RNAi of a subset of the five genes. Note CGH-1 granules (green) are detected after RNAi of K08F4.1, but not *str-82* or *dnj-17*. Large GLH-1 granules (red) are not detected after RNAi of any of the three genes shown. Quantitative data for all five genes are summarized in Table S2. GFP, Green fluorescent protein; RNAi, RNA interference; RNP, ribonucleoprotein.

Having identified a number of genes that promote MEX-3 granule assembly, we were interested to know if a failure to assemble MEX-3 granules when fertilization is delayed contributes to decreased oocyte quality, and developed an assay to determine if a correlation between the two exists. Our prediction was that if arrested oocytes do not assemble RNP granules, then oocyte quality will decrease, which may be revealed by embryonic lethality after mating. Because the phenotypes of many of our positive hits include embryonic lethality, we avoided those genes for this assay. Prior to mating into each female, we counted the number of arrested oocytes and noted if GFP::MEX-3 distribution was cytoplasmic or in granules. In control vector(RNAi) *fog-2* females that assemble GFP::MEX-3 granules in oocytes, > 90% of the fertilized embryos hatch (Jud *et al.* 2008; Andux and Ellis 2008). We considered the percentage of embryos that hatched into viable larvae as a proxy for the quality of oocytes that had been arrested. We selected seven genes for which embryonic lethality has not been reported and first did RNAi in wild-type worms to ensure no essential roles in embryonic viability. More than 90% of oocytes gave rise to viable embryos for each gene (Figure 2A, blue bars). After RNAi in the *fog-2*;GFP::MEX-3 strain, we categorized each female based on whether GFP::MEX-3 granule assembly was disrupted, mated the female with a male, and determined the percentage of fertilized oocytes that hatched in each category. For five of the seven genes, oocyte quality was significantly reduced when MEX-3 granules did not assemble by up to 35% for *dnj-17* (comparing red to green bars); the exceptions were *cec-6* and R06A4.2 (Figure 2A). These results correlate a failure to assemble MEX-3 granules in arrested oocytes with reduced oocyte quality, and are consistent with the hypothesis that the assembly of RNP granules helps maintain middle-aged oocyte quality when fertilization is delayed.

The reduction in oocyte quality could be independent of the RNP granule assembly defect, it could result specifically from the absence of MEX-3 from the RNP granules, or it could result from more global aberrations in RNP granule assembly. To determine if the assembly of other RNP granule components is disrupted after RNAi of the five genes, we first examined the distribution of a P body protein, CGH-1 (Jud *et al.* 2008; Beshore *et al.* 2011). We found that, in contrast to the > 90% of control worms in which CGH-1 is detected in large cortical granules, after RNAi of the five genes (*str-82*, F13C5.3, W03C9.5, K08F4.1, and *dnj-17*), CGH-1 granules failed to assemble in 12–50% of worms. Overall, the CGH-1 phenotype was less penetrant than MEX-3, and in the case of K08F4.1, CGH-1 closely resembled the control (Figure 2B and Table S2). We also assayed the distribution of a germ granule protein, GLH-1, and found that GLH-1 granules failed to assemble after RNAi of the five genes in 26–69% of worms (Figure 2B and Table S2), and was also overall less penetrant than the MEX-3 phenotype. Thus, the underlying reason for decreased oocyte quality may not be specific to MEX-3, since in several cases where oocyte quality appears to be decreased, multiple proteins fail to form RNP granules normally. However, the fact that some MEX-3 regulators did not appear to be required for CGH-1 or GLH-1 localization to granules suggests the possibility of at least some protein-specific regulators of condensation.

### Role of IDR proteins in RNP granule assembly

**Figure 3:**
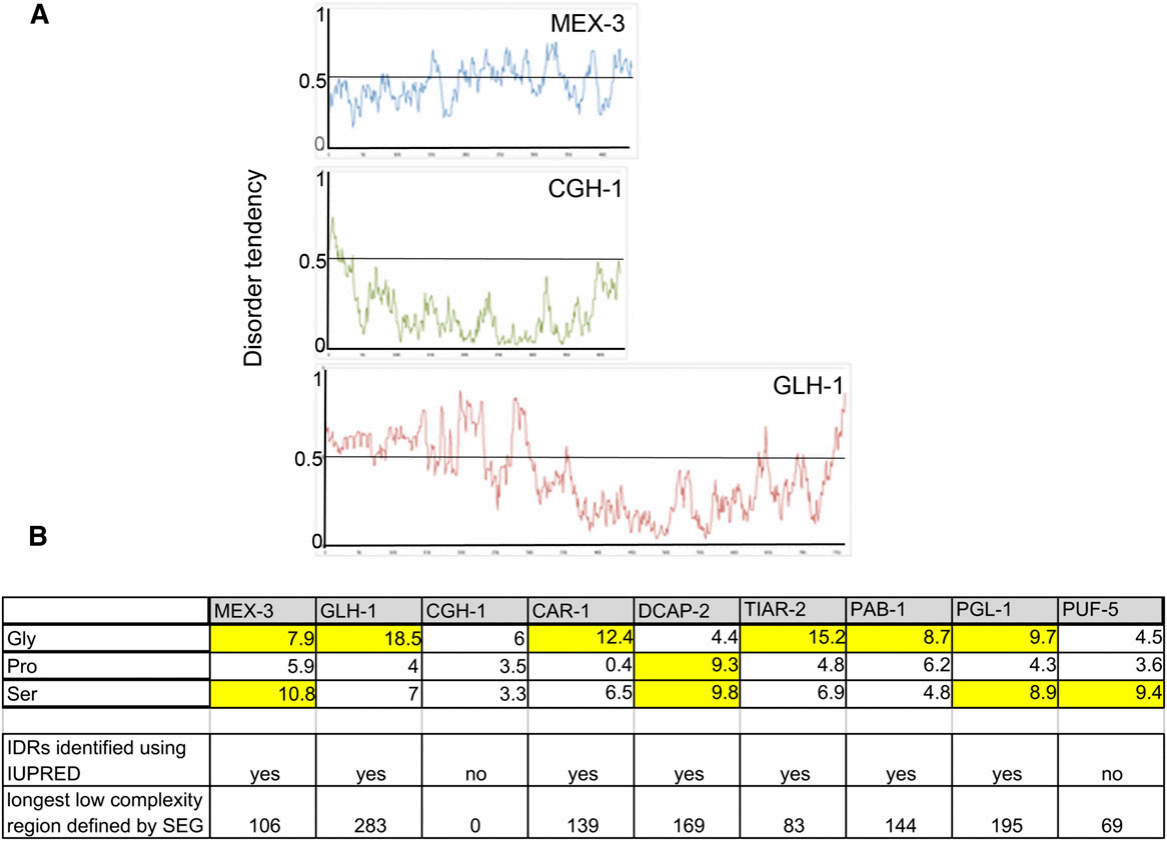
RNP granule components include many proteins with IDRs. (A) Graphs showing disorder tendencies of sequences along each protein [calculated using IUPRED and long disorder parameters; http://iupred.enzim.hu/ (Dosztanyi *et al.* 2005)]. Scores > 0.5 indicate disorder. MEX-3 and GLH-1 have large stretches of IDRs; in contrast, CGH-1 is a mostly ordered protein. (B) Overrepresentation of disorder-inducing amino acids in subset of nine RNP granule proteins. Percentages highlighted in yellow are overrepresented (Ferron *et al.* 2006). Seven of nine proteins contain IDRs as identified by IUPRED, and eight of nine have a low complexity region identified by the SEG server. Gly, glycine; IDRs, intrinsically disordered regions; Pro, proline; RNP, ribonucleoprotein; Ser, serine.

IDRs of RNA binding proteins can promote phase separation and the formation of liquid droplets *in vitro*, and in some limited studies, IDRs appear to contribute to the formation of RNP granules *in vivo* (Gilks *et al.* 2004; Decker *et al.* 2007; Lee *et al.* 2013; Updike *et al.* 2011; Wang *et al.* 2014; Elbaum-Garfinkle *et al.* 2015). In seeking to understand the regulation of RNP granule assembly in oocytes, we asked how many protein components of RNP granules in arrested oocytes contain IDRs. We first examined the sequences of MEX-3, CGH-1, and GLH-1 to see if any differences correlate with the differences observed among the immunofluorescence data (Figure 2 and Table S2). MEX-3 has several IDRs, identified using IUPRED (Dosztanyi *et al.* 2005), the longest of which is also identified as a low complexity region, as defined using SEG (http://mendel.imp.ac.at/METHODS/seg.server.html, Wootton 1994) (Figure 3, A and B). MEX-3 is also overrepresented in serine residues (10.8%) (Figure 3B), which are one of three disorder-inducing amino acids, along with glycine and proline (Ferron *et al.* 2006). GLH-1 also has multiple IDRs in its N-terminal half, as well as a 283 amino acid region of low complexity, 7% serine residues, and 18.5% glycine residues (Figure 3, A and B). In contrast, CGH-1 appears to be a mostly ordered protein, with no regions of low complexity and no overrepresented residues that promote disorder (Figure 3, A and B). While speculative, it is possible that different biochemical characteristics of CGH-1 may contribute to it being regulated differently than MEX-3 and GLH-1 with regards to phase separation properties. We also examined the profiles of six other protein components of RNP granules, and found that all of the proteins except PUF-5 have IDRs, low complexity regions, and at least one overrepresented disorder-inducing residue (Figure 3B and Figure S1). While PUF-5 has one short region of low complexity, it has no predicted IDRs. Overall, our survey of *in vivo* RNP granule components is consistent with the *in vitro* studies that suggest a prominent role for IDRs in promoting liquid–liquid phase separation (LLPS), *i.e.*, assembly of RNP granules. The precise roles of IDR proteins in recruiting heterotypic IDRs and in regulating the dynamics of RNP remodeling in oocytes remain to be determined.

**Figure 4:**
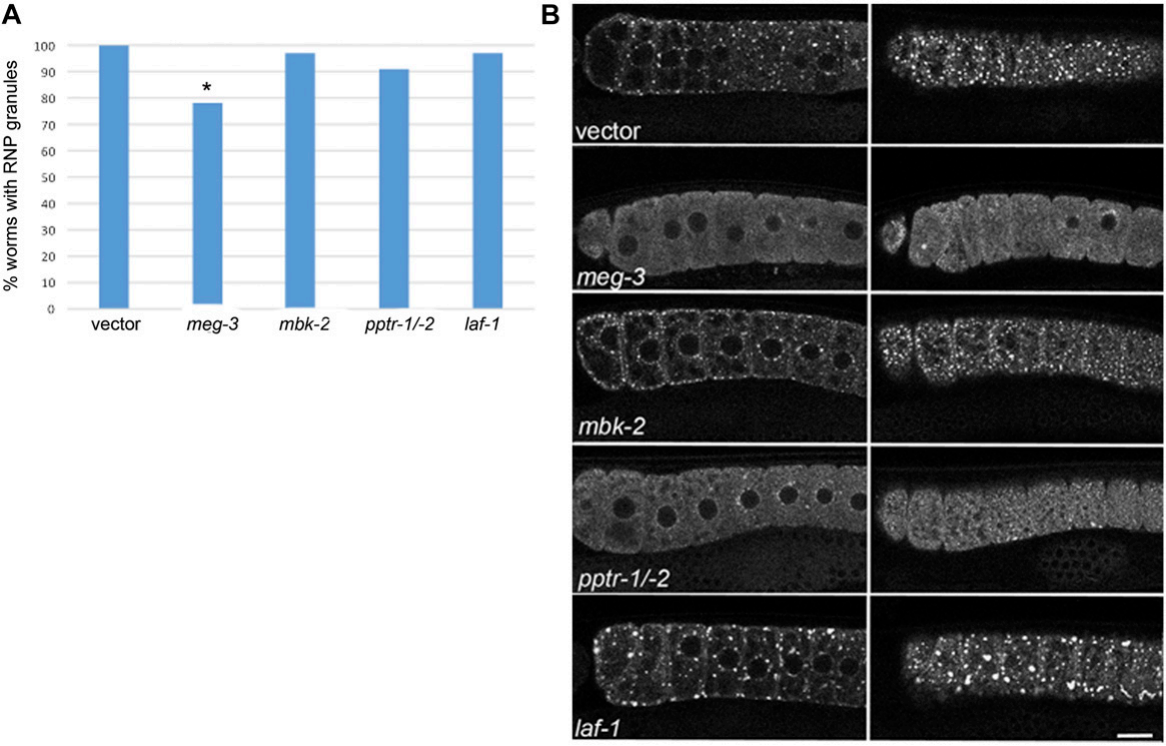
Role of IDR proteins in regulating oocyte RNP granules. (A) MEG-3 appears to promote the assembly of RNP granules in oocytes; however, LAF-1 does not appear to be required. No significant differences were observed for *mbk*-2 or *pptr-1/*-2 in terms of the percent of germ lines with RNP granules. A minimum of 22 germ lines were examined for each gene; * *P* < 0.05 by Fisher’s exact test. (B) Live images of GFP::MEX-3 after RNAi of IDR proteins in a *fog-2* background. Images on the left are midfocal views of proximal germ lines, and images on the right are cortical views of the same germ lines. MEG-3 appears to promote GFP::MEX-3 granule assembly in arrested oocytes. The phenotype of few RNP granules and high levels of diffuse GFP after *pptr-1/-2* RNAi was not penetrant; however, it was never observed in the vector control. In contrast to MEG-3, LAF-1 may function in an opposite manner and repress the assembly of large granules in arrested oocytes. RNAi of *laf-1* resulted in a 20% increase in the percent of germ lines with large RNP granules (> 5 μm) enriched at the cortex; compare *laf-1* cortical view to vector control cortical view. Image J was used for quantitation (data not shown). GFP, green fluorescent protein; IDRs, intrinsically disordered regions; RNP, ribonucleoprotein.

We next addressed whether either of two IDR proteins that modulate embryonic germ granule assembly, LAF-1 and the MEG proteins (Wang *et al.* 2014; Elbaum-Garfinkle *et al.* 2015), also regulate RNP granules in arrested oocytes. We exposed worms to RNAi at the L1 stage of development, consistent with our screen methods, and also at the L4 stage, consistent with methods used to examine embryo phenotypes. RNAi of *meg-3* at the L1 or L4 stage resulted in decreased RNP granule assembly in oocytes (Figure 4, A and B, reduced to 78%, compared to 100% in controls, *P* < 0.05). Since *mbk-2* and *pptr-1/-2* regulate the phosphorylation state of the MEG proteins and modulate embryonic germ granules (Wang *et al.* 2014), we next asked if this kinase and phosphatase regulate oocyte RNP granules. Since RNP granules were detected in 97% of germ lines after RNAi of *mbk-2* (Figure 4, A and B), we next used ImageJ to quantitate the number of granules in the most proximal oocytes and observed a modest increase in the average number of total granules, 10% higher compared to control worms (data not shown). This result is similar to the increased number of germ granules seen in *mbk-2* embryos, though less penetrant, and suggests a possible role for MBK-2 in disassembly of granules in oocytes. In embryos, the phosphatase PP2A promotes assembly of germ granules. After RNAi of *pptr-1/-2*, we observed RNP granule assembly in 91% of germ lines (Figure 4, A and B). The 9% of worms failing to assemble RNP granules normally was not statistically different from the control; however, the low penetrance may reflect the difficulty in using RNAi to effectively knock down gene expression of two genes simultaneously, or that PP2A does not play the same role in regulating RNP granules in oocytes as in embryos. We next examined the role of the intrinsically disordered protein LAF-1. In contrast to the role of *laf-1* in promoting germ granules in embryos, we found that after *laf-1*(RNAi), RNP granule assembly occurred efficiently in 97% of germ lines (Figure 4A). Surprisingly, the granules appeared slightly larger than in control oocytes, which was especially apparent in cortical focal planes (Figure 4B, right column). We next used Image J to quantify the percent of worms with very large granules, > 5 μm in diameter. While RNP granules are consistently detected in 100% of control, vector(RNAi) worms, very large granules > 5 μm are seen in 74% of control worms. RNAi of *laf-1* resulted in a 20% increase in the percent of germ lines with large RNP granules enriched at the cortex, and the number of granules > 5 μm per *laf-1* germ line was 4.5, compared to 2.2 in the vector control (data not shown). Therefore, *laf-1* may have an opposite function in modulating RNP granules in oocytes, as compared to embryos. Taken together, these results suggest that at least two IDR proteins that regulate embryonic germ granules also regulate the assembly of large RNP granules in arrested oocytes. While MEG-3 and its regulators appear to have similar roles at both times of development, LAF-1 may have an opposite role in arrested oocytes compared to the early embryo, suggesting unique regulators of different RNP granules.

### Regulators of different RNP granules suggest granule-specific controls

The composition of the RNP granules in arrested oocytes includes a subset of mRNAs and proteins associated with the smaller germ granules that associate with germ cells throughout development (Schisa *et al.* 2001; Jud *et al.* 2008; Noble *et al.* 2008). We asked how many of the positive regulators of large RNP granules we identified matched the 173 regulators of germ granules (Updike and Strome 2009). Somewhat surprisingly, only six genes were in common (*zen-4*, *tbg-1*, *rnp-4*, *atx-2*, *csr-1*, and *cyp-31A3*); moreover, several of the largest gene classes we observed, germ plasm, the cell cortex, the cytoskeleton, chromosome segregation, and RNAi, were not identified as germ granule regulators. The low number of genes in common could arise for a variety of reasons. The germ granule screen was performed using a different marker, PGL-1 *vs.* MEX-3, and with different RNAi conditions, exposing larvae at L3/L4 stage *vs.* L1 stage. In addition, the majority of genes identified in the PGL-1 screen had phenotypes in F1 embryos (157) rather than in P0 germ lines (16), and they included a variety of PGL-1 phenotypes, not only a failure of PGL-1 to localize normally to germ granules. Perhaps the most interesting explanation for the lack of overlap among the regulators is the possibility that embryonic P granules differ fundamentally from RNP granules in the germ line. This idea is supported by the fact that several protein components of embryonic P granules are not detected in P granules in oocytes or the adult germ line (Updike and Strome 2009; Schisa 2012; Voronina 2013). Moreover, in a recent screen for modifiers of solid RNP granules in *cgh-1* oocytes, very little overlap was observed with P granule regulators (Hubstenberger *et al.* 2015).

Interestingly, only modest overlap was seen between regulators of liquid RNP granules in arrested oocytes and solid RNP granules in *cgh-1* oocytes. Of the gene classes we identified, several are notable as not being identified in the screen for modifiers of solid granules, including the cell cortex, the cytoskeleton, chromosome segregation, and RNAi. In addition, of 66 recently identified modifiers of solid granules, only 20 genes also promote liquid RNP granule condensation/assembly in *fog-2*-arrested oocytes (Hubstenberger *et al.* 2015). Our screen identified three of these twenty genes, *car-1*, *atx-2*, and *puf-5*, and some of the same, unexpected gene families, including the TRiC chaperonin complex (*cct-4*) and chaperone proteins in the DnaJ family (*dnj-17* and *dnj-22*, Table S1). Our results support the notion that protein folding regulates RNP remodeling, as suggested by the recent identification of *cct-5*, *cct-6*, and *dnj-23* (Hubstenberger *et al.* 2015), the identification of *cct-2*, *cct-3*, *cct-7*, and *cct-8* (Updike and Strome 2009), and the evidence in yeast that CCT subunits modulate P body formation (Nadler-Holly *et al.* 2012). Reasons we did not identify all 20 genes include the possibility of false negatives, the use of GFP::MEX-3 instead of GFP::CAR-1 as a marker, and/or applying RNAi at the L1 instead of L3/L4 stages. Overall, the differences in gene classes suggest the possibility that at least a subset of cellular pathways required for the assembly of RNP granules after meiotic arrest are distinct from those that modulate the polymerization of solid granules.

Taken together, the lack of similarity observed among regulators of germ granules, liquid RNP granules in arrested oocytes, and solid granules in *cgh-1* oocytes, supports the notion of fundamental differences in the regulation of RNP granule phase transitions during meiotic arrest. Distinct pathways may be needed to modulate different RNP granule types due to different phase transitions and/or developmental timing events.

### Summary

In our screen to identify regulators of RNP granule assembly, we identified a large number of gene knockdowns that disrupt the assembly of GFP::MEX-3 granules to variable degrees. Since arrested oocytes that fail to localize MEX-3 into cortical granules were correlated with reduced oocyte quality, our choice of marker protein appears to be reasonable to gain insights into RNP granule function. In addition to some of the expected categories, we identified multiple genes in the novel cell component classes of cell cortex, ER, cytoskeleton, and nuclear chromosome. To complement the RNAi screen, we investigated the role of IDR proteins in regulating the assembly of RNP granules in *C. elegans* oocytes. We found that seven of nine proteins have IDRs; CGH-1 lacks IDRs and appears to be regulated at least somewhat independently from two proteins with IDRs, MEX-3 and GLH-1. Of two IDR regulators of germ granule assembly in early embryos, MEG-3 modulates MEX-3 condensation, but LAF-1 does not appear to be required for this function. This mixed result is indicative of our larger-scale comparison of regulators of germ granules, RNP granules in meiotically-arrested oocytes, and solid RNP granules in *cgh-1* oocytes, which reveals little overlap among regulators and suggests granule-specific controls may be required due to differential developmental timing or phase transition differences.

## Supplementary Material

Supplemental Material
