# Supplementary material for: RNAi Screen Identifies Novel Regulators of RNP Granules in the Caenorhabditis elegans Germ Line
Source: G3 (Bethesda). 2016 Jun 9;6(8):2643–54. doi: 10.1534/g3.116.031559 (PMC4978917; doi:10.1534/g3.116.031559)
Supplement: Supplemental Material [file supp_6_8_2643__index.html]

RNAi Screen Identifies Novel Regulators of RNP Granules in the Caenorhabditis elegans Germ Line — Supplemental Material 

# RNAi Screen Identifies Novel Regulators of RNP Granules in the *Caenorhabditis elegans* Germ Line

## Supplemental Material for Wood *et al.*, 2016

**Files in this Data Supplement:**

- Figure S1 - The majority of RNP granule protein components have IDRs. (.pdf, 294 KB)
- Table S1 - List of genes identified as regulators of RNP granule assembly in arrested oocytes. (.xlsx, 25 KB)
- Table S2 - Percent of worms in which large granules are not detected by the indicated protein marker. (.xlsx, 35 KB)
