# Supplementary material for: RNAi Screen Identifies Novel Regulators of RNP Granules in the Caenorhabditis elegans Germ Line
Source: G3 (Bethesda). 2016 Jun 9;6(8):2643–54. doi: 10.1534/g3.116.031559 (PMC4978917; doi:10.1534/g3.116.031559)
Supplement: Supplemental Material [file supp_g3.116.031559_FigureS1.pdf]

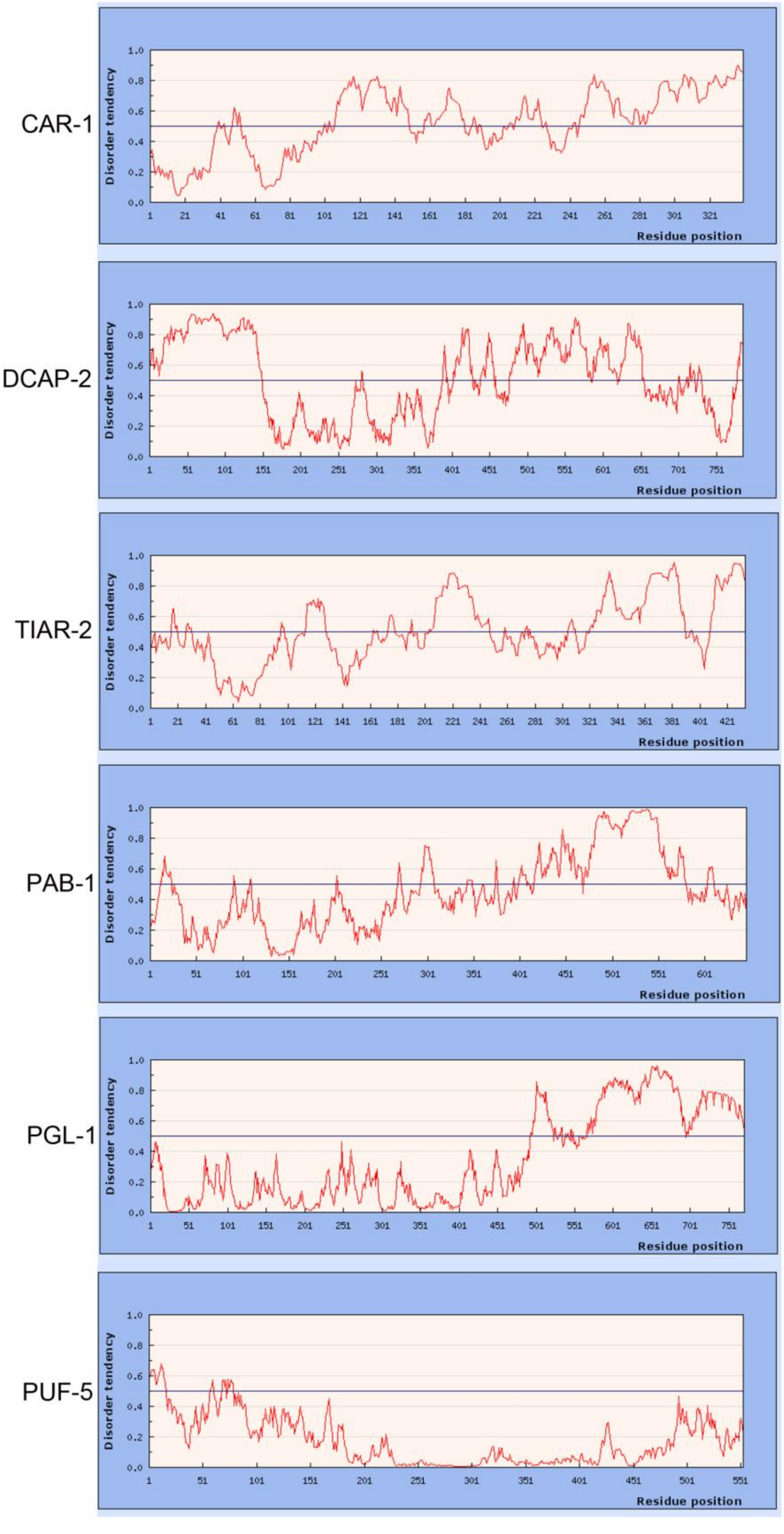

Figure S1. The majority of RNP granule protein components have IDRs. IDRs were identified using IUPRED (Dosztanyi et al. 2005). The disorder tendency was >0.5 for at least one long sequence in CAR-1, DCAP-2, TIAR-2, PAB-1, and PGL-1, but not in PUF-5.
